# Gut Microbiota, Deranged Immunity, and Hepatocellular Carcinoma

**DOI:** 10.3390/biomedicines12081797

**Published:** 2024-08-07

**Authors:** Emidio Scarpellini, Giuseppe Guido Maria Scarlata, Valeria Santori, Marialaura Scarcella, Nazarii Kobyliak, Ludovico Abenavoli

**Affiliations:** 1 Translational Research Center in Gastro-Intestinal Disorders (T.A.R.G.I.D.), Gasthuisberg University Hospital, KU Leuven, Herestraat 49, 3000 Lueven, Belgium; 2 Department of Health Sciences, University “Magna Graecia”, 88100 Catanzaro, Italy; giuseppeguidomaria.scarlata@unicz.it (G.G.M.S.); l.abenavoli@unicz.it (L.A.); 3 Gastroenterology Clinic, University of Padua, 35128 Padua, Italy; valeria.santori@studenti.unipd.it; 4 Anesthesia, Intensive Care and Nutritional Science—Azienda Ospedaliera “Santa Maria”, Via Tristano di Joannuccio, 05100 Terni, Italy; m.scarcella@aospterni.it; 5 Endocrinology Department, Bogomolets National Medical University, 01024 Kyiv, Ukraine; nazariikobyliak@gmail.com; 6 Medical Laboratory CSD, 02000 Kyiv, Ukraine

**Keywords:** gut microbiota, immune system, liver cancer, hepatocellular carcinoma, checkpoint inhibitors

## Abstract

Background: Liver cancer, particularly hepatocellular carcinoma (HCC), is a significant gastrointestinal disease with a mortality rate as high as nearly 80% within five years. The disease’s pathophysiology involves deranged immune responses and bile acid metabolism, with the gut microbiota (GM) playing a crucial role. Recent research highlights the potential of GM in influencing HCC treatment outcomes, especially regarding immune checkpoint inhibitors (ICIs). However, few patients currently benefit from ICIs due to a lack of effective response biomarkers. Aims and methods: This review aimed to explore the literature on HCC treatment issues, focusing on immune response, bile acid metabolism, and GM dysbiosis. This review included studies from PubMed, Medline, and major gastroenterology and hepatology meetings, using keywords like gut microbiota, immune system, liver cancer, and checkpoint inhibitors. Results: GM dysbiosis significantly impacts immune response and bile acid metabolism, making it a promising biomarker for ICI response. Modulating GM can enhance ICI treatment efficacy, although more research is needed to confirm its direct therapeutic benefits for HCC. Conclusions: GM dysbiosis is integral to liver cancer pathogenesis and treatment response. Its modulation offers promising therapeutic avenues for improving HCC prognosis and response to immunotherapy.

## 1. Introduction

Liver cancer is the second most frequent gastrointestinal cancer for mortality (with 830,180 related deaths worldwide in 2020) [1]. The most common type of primary liver cancer is hepatocellular carcinoma (HCC), which accounts for almost 90% of the cases. According to GLOBOCAN, HCC represents the fourth leading cause of cancer-related death, with a five-year survival rate of 18% for patients in an advanced stage [2].

HCC usually stems from chronic liver disorders, such as liver cirrhosis of both viral and non-viral origin [3]. The latter origin is vastly represented by metabolic-dysfunction-associated steatotic liver disease (MASLD) and, to a lesser extent, by autoimmune and primary liver diseases (namely, primitive biliary cholangiopathies and sclerosing cholangitis). A peculiarity of MASLD is its capability to develop HCC during its intermediate stage, namely metabolic-dysfunction-associated steatohepatitis (MASH) [4]. Thus, MASH patients’ surveillance is a critical step in patient management. HCC pathophysiology is complex: there is only limited knowledge about genome mutations in the frame of its pathophysiology. In fact, it involves direct liver damage by viruses, fat deposition, inflammation, oxidative stress, and alcohol, leading to a leaky gut with the passage of gut dysbiosis antigens and metabolites [5]. The subsequent chronic liver inflammation leads to fibrosis deposition until the onset of cirrhosis. Within this environment, the accumulation of genetic and epigenetic alterations over time results in HCC development. Interestingly, the role of the tumor immune microenvironment, characterized by “tolerance” towards mutating cells, has paved the way for the potential use of immunotherapy in clinical settings [6]. 

Indeed, early stages of HCC (according to the Barcelona Clinic Liver Cancer (BCLC) classification) benefit from curative orthotopic liver transplantation and/or surgical resection [7]. However, a high percentage of HCC patients are not eligible for these treatments, due to organ shortage and, most commonly, due to the advanced stage of the cancer, perhaps in a context of deranged chronic liver disease [8]. Therefore, percutaneous ablative therapies (namely, radiofrequency ablation and microwave ablation) are considered the first treatment approach in early and very early HCC stages. Their benefits include a survival time similar to surgery/transplantation, less invasiveness, and lower costs [7,8]. Unfortunately, they only have a local anti-tumor effect and do not control tumor burden [6,7,8]. Furthermore, the vast majority of HCC patients (almost 70%) are diagnosed in the intermediate (B) or advanced (C) stages and can be considered for transarterial/systemic treatments. They can be considered non-curative or “palliative” [9]. Looking at systemic treatments, the multikinase inhibitor sorafenib has been used as the first-line therapy for Child–Pugh A liver cirrhosis and unresectable/metastatic HCC: survival time was about 3 months longer than those of patients who received placebo [10]. Subsequently, Lenvatinib was approved as an alternative to sorafenib because of its non-inferiority [7,8]. Multitarget tyrosine inhibitors (namely regorafenib and cabozantinib) [11] and vascular endothelial growth factor (VEGF) receptor inhibitors (ramucirumab) are single-agent second-line treatments for patients not responding to sorafenib [11]. To date, the combination of atezolizumab/bevacizumab is the standard first-line treatment for patients with advanced HCC [8,12]. In the milieu of the tumor-deranged immune microenvironment, immune checkpoint inhibitors (ICIs) have emerged as alternatives for patients with adequate performance status. Specifically, in 2017, the FDA approved nivolumab as an add-on treatment for patients failing to respond to sorafenib. Subsequently, pembrolizumab was used. These molecules are programmed cell death protein-1 (PD-1) inhibitors. More recently, the combination of ipilimumab (cytotoxic T-lymphocyte-associated protein 4 inhibitor) and nivolumab has also been used. Overall, ICIs are superior to sorafenib in terms of overall survival (OS) and progression-free survival [6,7,8]. However, the crude reality of data reminds clinicians and researchers that almost 60% of HCC patients (10–20% for first-line and 10% for second-line) do not respond to ICIs. Thus, we need effective biomarkers of treatment-response prediction.

Gut dysbiosis assessment via newer metagenomic techniques is an emerging item in the field [13]. From a more general point of view, the criteria for predicting prognoses and deciding treatment modalities for BCLC are not always in line with patients’ “personalized” therapeutic approach. For this reason, several pieces of evidence from the recent literature are in favor of a novel approach, namely “converse therapeutic hierarchy” [14]. The latter consists of the “personalized” integration of advanced treatment modalities over previous rigid hierarchic flow-chart running.

Thus, we aimed to review literature data on the definition and composition of gut microbiota in a healthy state and during liver cancer, its interaction with the immune system in HCC patients, and its potential role as a biomarker, highlighting its future therapeutic implications.

## 2. Materials and Methods

We conducted a search on PubMed and Medline for original articles, reviews, meta-analyses, and case series using the following keywords, their acronyms, and their associations: gut microbiota, immune system, liver cancer, hepatocellular carcinoma, checkpoint inhibitors. When appropriate, preliminary evidence from abstracts belonging to main national and international hepatological and gastroenterological meetings (e.g., Italian association for liver disease study, European association for the study of liver disease, United European Gastroenterology Week, Digestive Disease Week) was also included. The papers found from the above-mentioned sources were reviewed by two of the authors (E.S. and L.A). The last Medline search was dated 30 April 2024.

## 3. Results

### 3.1. Current Medical and Therapeutic Umnet Needs in HCC Patients

Although the treatment paradigm of HCC has undergone a real revolution after 2017, the use of ICIs, alone or association with targeted ones, has encountered several drawbacks. For example, HCC patients differ greatly from each other, only a few genetic mutations are currently known, and there is a lack of effective biomarkers [15]. Regarding druggable mutations, the main known ones are only TERT, CTNNB1 (WNT/β-catenin), and TP53 [16]. Therefore, there are two main needs: screening for highly mutated and druggable targets and drugging the existing targets that have been discovered with new paths towards interfering mutated genes [16]. Indeed, promising results have opened the way for an advancement in HCC treatment. In a preclinical study, silencing TERT expression with antisense oligonucleotides led to the inhibition of tumor growth in cell lines and animal models [17]. In addition, nonsteroidal anti-inflammatory agents like celecoxib and sulindac can inhibit the WNT/β-catenin signaling pathway in HCC cell lines [18]. The overexpression of wild-type AXIN1 is able to inhibit the proliferation of HCC cells. Thus, it can induce and anticipate the programmed cell death of tumor cells [19]. In preclinical studies, salinomycin and NVP-TNKS656 have been successfully targeted by the upstream molecules of the WNT/β-catenin signaling pathway [20]. Finally, ambitious efforts to target the p53 pathway are underway [21]. PD-1, PD-L1, CD3, and CD8 are considered as possessing predictive power on the response efficacy of ICIs. However, PD-L1 has been found to not be a reliable biomarker [22]. In fact, nivolumab could retrieve the treatment response irrespective of tumor PD-L1 expression [23]. However, the evidence is heterogenous: in the KEYNOTE-224 trial, some patients responded to pembrolizumab according to PD-L1 expression [24]. Reasons for such a variability in treatment-response assessment can be explained by PDL1 expression. This is regulated by multiple pathways, such as the tumor cell life cycle, and also by the inflammatory response killing the cancer cells within the tumor microenvironment. PD-L1 expression is one feature of the last process [24]. Intriguingly and paradoxically, Pfister et al. showed that anti-PD-1 therapy promotes the progression of NASH-induced HCC. In detail, the treatment activated CD8+PD1+ T cells within the tumor and the number and size of tumor nodules grew. Interestingly, this increase was prevented by either CD8+ T cell depletion or TNF neutralization [25]. There are also biomarkers for KI drug selection: mutations in the PI3K-AKT-mTOR pathway, which have been associated with lower cases of treatment response to Sorafenib. Moreover, VEGF, ANG2, FGF21, and FGFR4 immunostaining positivity has been associated with the treatment response to Lenvatinib [22,23,24,25]. In more recent years, a novel approach to finding HCC biomarkers entails looking for the co-localization of biomarkers, based on subsiding molecular mechanisms. As an example, Ankur Sharma et al. employed scRNA sequencing and revealed a shared immunosuppressive oncofetal ecosystem shared by the fetal liver and HCC (namely, characterized by the enrichment of Tregs and exhausted CD8+T cells). Subsequently, VEGF and NOTCH signaling have been found to contribute to the maintenance of the immunosuppressive fetal cancer ecosystem [26]. This finding has been reinforced by the excellent performance of VEGF inhibitors in patients with HCC. Challenges and promising results regarding medical and therapeutic needs in HCC patients are summarized in Table 1.

### 3.2. Gut Microbiota Composition in Healthy Subjects and Liver Cancer Patients

The human gut microbiota consists of a complex ecosystem of bacteria, viruses, archaea, protozoa, yeasts, and fungi inhabiting the gut. This ecosystem can play a role in several metabolic and immune system processes [27]. The first functions of the gut microbiota to be studied have been digestion and nutrient absorption. There is a fine commensal relationship among gut microbes and humans [28]. The main components of bacterial gut microbiota are strict anaerobes and, to a lesser extent, facultative anaerobes and aerobes. Thus, *Bacteroidetes* and *Firmicutes* are the most common out of more than 50 bacterial phyla in the human gut [27,28]. The most typical pattern of altered composition of the gut microbiota, also known as “dysbiosis”, is usually observed in patients with liver cirrhosis and HCC. Meta-analysis data by Huang et al. from 17 studies revealed an increased abundance of genera *Enterobacter* and *Enterococcus* spp. and a decreased abundance of genera *Lactobacillus* and *Bifidobacterium* spp. in cirrhotic patients [29]. Furthermore, in their study on gut dysbiosis, Ponziani et al. differentiated between NAFLD-related liver cirrhosis with HCC, NAFLD-related liver cirrhosis without HCC, and healthy controls. Interestingly, they showed an increased abundance of *Lactobacillus*, *Haemophilus*, *Enterococcus,* and *Klebsiella* genera and a reduced abundance of *Akkermansia* and *Methanobrevibacter* genera in cirrhotic patients vs. healthy controls [30]. To note, liver cirrhosis stage was uniform as the enrolled patients all belonged to the Child A class according to the Child–Pugh liver cirrhosis stage classification. Specifically, the assessment showed that HCC patients had an increased abundance of *Bacteroides*, *Enterococcus*, and *Ruminococcaceae* genera and a reduced abundance of *Bifidobacterium* spp. vs. cirrhotic without HCC evidence. Zhang et al. stratified HCC patients according to their BCLC status in three stage groups: those with an advanced HCC stage showed increased concentrations of *Enterococcus* and *Enterobacteriaceae* and decreased concentrations of *Actinobacteria* and *Bifidobacterium* genera [31]. From a mechanistic point of view, Ren et al. discovered decreased levels of butyrate-producing bacteria (e.g., *Ruminococcus*, *Oscillibacter*, and *Faecalibacterium* genera) and increased levels of lipopolysaccharide (LPS)-producing bacteria (e.g., *Klebsiella* and *Haemophilus* genera) in HCC patients vs. cirrhotic and healthy subjects [32]. Indeed, liver cirrhosis patients had significantly lower microbial diversity vs. both HCC and healthy subjects. Similarly, enrolled cirrhotic patients fell within the Child A class. Differentiating the cohort of patients even more, Zheng et al. enrolled patients with hepatitis, liver cirrhosis, cirrhosis-related HCC, non-cirrhosis-related HCC, and healthy individuals. Increased levels of the *Bacteroidetes* and *Fusobacteria* class and gut microbiota diversity were recorded in HCC patients vs. other groups [33]. Of distinction, no significant difference in gut dysbiosis was observed in HCC patients despite their different etiology. Yan et al. involved patients with HBV-related liver cirrhosis, HBV-related HCC, and healthy volunteers: an increased abundance of pro-inflammatory bacteria (e.g., *Proteus*, *Klebsiella*, and *Streptococcus* genera) and a reduced abundance of butyrate-producing bacteria (e.g., *Bacteroides* phylum and *Firmicutes* species) were found in the gut of HBV-related liver cirrhosis and HBV-HCC patients vs. healthy subjects [34]. Interestingly, gut microbiota diversity decreased in HBV-HCC patients vs. findings by Ren et al. This apparent discrepancy can be explained by the different sample size, data analysis, and, last but not least, the 16S ribosomal RNA sequencing of fecal microbiota, which is not devoid of bias as novel metagenomic methods [35]. Intriguingly, another apparently contradictory finding was that *Lactobacillus* abundance was found to be increased in HBV-related HCC patients. This can be explained by a more severe cancer-derived inflammatory environment. Finally, of consideration is the composition of liver cirrhosis patients: Child A patients made up 36% of enrolled subjects, Child B made up 47%, and the remaining percentage was composed of Child C patients. The different and variegate composition of the study population could have created differences in the results. In fact, Bajaj et al. studied the ratio of autochthonous vs. non-autochthonous taxa (namely, the cirrhosis dysbiosis ratio (CDR)) in different disease stages. In detail, 219 cirrhotics (121 compensated outpatients, 54 decompensated outpatients, 44 inpatients) and 25 age-matched healthy controls were enrolled. CDR was highest in healthy subjects (2.05) vs. compensated (0.89) and decompensated (0.66) patients. More in detail, MELD score was negatively correlated with *Clostridiales XIV*, *Lachnospiraceae,* and *Ruminococcaceae* (r = −0.3, *p* < 0.0001 for all) and with *Rikenellaceae* (r = −0.2, *p* < 0.0001), while it was positively correlated with potentially pathogenic taxa (namely, *Staphylococcae* (r = 0.2, *p* = 0.03), *Enterococceae* (r = 0.4, *p* < 0.0001), and *Enterobacteriaceae* (r = 0.3, *p* = 0.001) [36] (Table 2).

Interestingly, liver patients without cirrhosis progression have different gut dysbioses: in non-alcoholic steatohepatitic (NASH) patients, there is an increased abundance of the Proteobacteria phylum and Prevotella and Porphyromonas species and a decreased abundance of Firmicutes species and the Bacteroidetes class; in alcoholic liver disease (ALD) patients, there is an increased abundance of the Prevotellaceae family and a decreased abundance of the Bacteroidaceae family; in chronic C hepatitis patients, there is an increased abundance of the Enterobacteriaceae genus and Bacterioidetes class and a decreased abundance of the Firmicutes species; in chronic B hepatitis patients, the abundance of the Bacteroidetes class is increased, while those of Firmicutes species is decreased [38,39].

### 3.3. Gut Microbiota and Liver Cancer Pathophysiology

#### 3.3.1. Gut Microbiota, Bile Acids, Immunity, and Liver Cancer Pathophysiology

**Figure 1:**
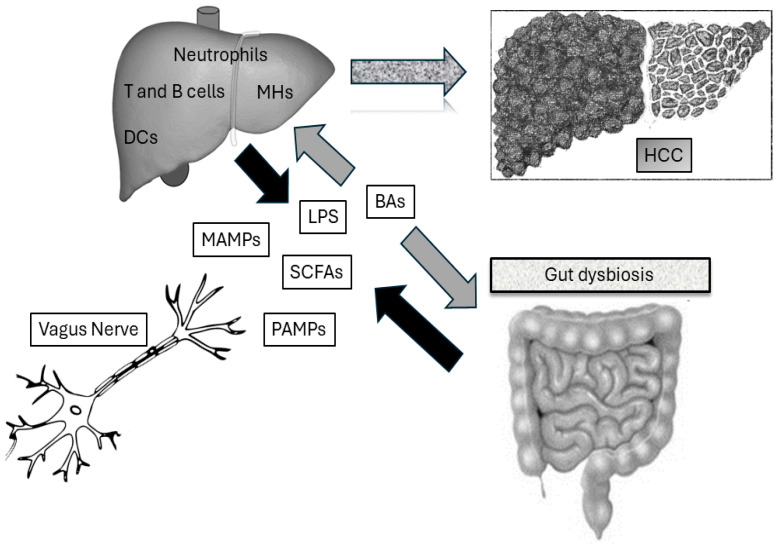
Mechanisms underlying the interactions between the gut microbiota, deranged immunity, and liver cancer. Gut microbiota dysbiosis drives immune system immunosurveillance impairment in the liver through its metabolites and the interaction with bile acid metabolism. Vagus nerve signaling modulates immune response within the liver and is affected by gut microbiota composition. LPS: lipopolysaccharide; SCFAs: short-chain fatty acids; MAMPs: microbe-associated molecular patterns; PAMPs: pathogen-associated molecular patterns; BAs: bile acids; DCs: dendritic cells; MHs: mesenchymal hepatic cells.

The gut–liver axis model explains the mechanistic connection between the gut microbiota, its particles, its metabolites, and the liver. An altered intestinal permeability allows the passage of molecules up to the portal circulation within the liver where they can affect hepatic immunity. This is the preamble to the development of liver cancer, in general, and especially of HCC [37]. In particular, liver sinusoidal endothelial cells (LSECs) “sense” the gut microbiota and induce Kupffer cells and lymphocytes to build up a protective response [40]. A disruption of immune surveillance favors liver inflammation [41]. Furthermore, chronic liver inflammation is a tumorigenic environment that is fed by several cytokines, chemokines, growth factors, prostaglandins, and pro-angiogenic factors [42]. The second step towards tumorigenesis is the imbalance and impairment of senescence surveillance by T cells on pre-malignant hepatocytes [43]. For example, in an ex vivo setup, the gut dysbiosis bacterial products from HCC patients added to the peripheral blood mononuclear cells of healthy subjects induced a switch towards the immunosuppression of the T cell phenotype. Specifically, this was caused by the clonal expansion of regulatory T cells (Tregs) and, conversely, by the attenuated development of cytotoxic CD8+ T cells [44]. In animal models of steatohepatitis-induced HCC, gut dysbiosis was associated with an elevated count of myeloid-derived suppressor cells (MDSCs) and, on the other hand, a significant reduction in CD4+ and CD8+ T cell abundance within the liver [30]. Likewise, human studies underlined and specifically showed that the abundance of specific bacterial strains (namely, the Bacteroides genus) correlated with increased MDSCs and the cytokines IL-8 and IL-13 in HCC patients. Indeed, cytokines affect MDSC recruitment and proliferation [45]. Corroborating the data, gene expression analysis of cirrhotic livers showed that bacterial species translocation into the liver correlates with the T cells’ exhaustion biomarkers: cytotoxic T lymphocyte-associated antigen 4, PD-1, and thymocyte-selection-associated HMG box. The resulting immunosuppression is an expression of impaired cancer surveillance [20]. Restoring gut eubiosis in liver cancer patients can lead to the re-establishment of immunosurveillance. Animal model studies have demonstrated that treating pathogenic bacteria with antibiotics can reverse immunosuppressive events that promote tumors. This results in a reduced tumor burden [20,46]. Short-chain fatty acids (SCFAs) and bile acids (BAs) are the two main gut microbial metabolites involved in HCC pathophysiology [47]. SCFAs (specifically, acetate, propionate, and butyrate) are produced by the fermentation of undigested carbohydrates, amino acids, lactic acid, and fibers [23]. They can modulate immune cell functioning through histone deacetylase inhibition and the activation of G protein-coupled receptors [24]. Interestingly, some data showed a decreased level of SCFA-producing bacteria in the feces of HCC patients (namely, *Lachnospira*, *Ruminococcus*, and *Butyricicoccus* bacteria genera) [48]. However, increased SCFA concentrations have been found in HCC patients [19]. Indeed, they could promote immunosuppression via microbiota antigen-specific T helper (Th)1 cell IL-10 production or via the suppression of inflammatory macrophages in the lamina propria. The latter would facilitate gut dysbiosis [49]. Indeed, the interaction and role of SCFA concentration and gut microbiota include other actors: a high inulin diet increases SCFA production, with consequent liver inflammation, neutrophil influx, and HCC development in mice with gut dysbiosis and elevated BAs and hyperbilirubinemia [50]. In fact, the level of liver injury is significantly reduced by the antibiotics’ modulation of fiber-fermenting bacteria [51]. In parallel, subjects with NAFLD-related HCC had enriched SCFA fecal concentrations and, accordingly, an increase in peripheral blood Tregs vs. cytotoxic CD8+ T cells subject to NAFLD-related HCC [23]. Altogether, these data confirm that gut dysbiosis, advanced liver disease, and increased SCFA production could promote an immunosuppressive environment allowing HCC outbreak. Finally, we can gather that SCFAs can have a beneficial role in chronic liver disease but that the degree of effectiveness depends on the stage of liver disease, the degree of dysbiosis, and, last but not least, the BA profile and concentration. Deranged BA metabolism associated with gut dysbiosis has been reported in HCC [52]. Primary BAs are synthesized from cholesterol within the liver (namely, cholic and chenodeoxycholic acid) [53]. Subsequently, these conjugate with taurine or glycine and are released into the duodenum, exposed to the gut microbiota, and metabolized into secondary BAs (namely, lithocholic acid and deoxycholic acid (LCA and DCA)). The fraction that is not reabsorbed towards the liver remains in systemic circulation. In the blood torrent, they act as signaling molecules. For example, they can activate nuclear receptors (mainly, the farnesoid X receptor (FXR) and Takeda G protein-coupled bile acid receptor 1) [29]. Importantly, FXR activation within the liver suppresses lipogenesis and increases lipolysis. This key step helps in preventing fat accumulation in hepatic cells. Of further importance, in liver-specific FXR-knockout mice, there is a significant 20% incidence of HCC [54]. In addition, the administration of obeticholic acid as an FXR agonist is able to downregulate STAT3, limiting cancer promotion [55]. Thus, we can assume that dysregulated BA accumulation and the suppression of FXR expression can contribute to liver carcinogenesis. Peculiarly, the balance between primary and secondary BA concentration adjusts those between tumor growth/anti-cancer immunomodulation [31]. In fact, both mice and human studies confirmed secondary BAs promoting an immunosuppressive environment for cancer cells. The gut microbiota regulates the metabolism of synthesis from primary to secondary BAs, regulating the C-X-C motif ligand 16 expression of LSECs and the migration of anti-tumor natural killer T cells to the liver [23,31]. Accordingly, elevated secondary BAs increase M2-like tumor-associated macrophage polarization within the cancer environment [56]. However, other literature evidence suggests that not all secondary BAs are pros for HCC formation. For instance, in a murine animal model, the secondary BAs 3-oxo-lithocholic acid and isolithocholic acid were able to inhibit Th17 expression [57]. Their concentration was phenotypically associated with better cancer prognosis [58]. More interestingly, HCC patients responding to immunotherapy had a higher concentration of fecal secondary BAs (namely, UDCA, tauro-UDCA, ursocholic acid (UCA), and murideoxycholic acid (MDCA)) vs. non-responders [59]. Consequently, diets modulating both SCFA and BA concentrations can influence HCC development. Indeed, diets rich in fermentable fiber are associated with HCC formation in animal models with cholestatic-induced liver damage. An increased SCFA concentration and high levels of BAs can reduce CD8+ T cells and increase Tregs and immunosuppressive immunoglobulin (Ig)A+ B cells within the liver [60]. In these studies, elevated serum BAs and hyperbilirubinemia concentrations were induced by high fermentable fibers. Thus, an imbalanced environment favoring excessive SCFA and BA production can lead to an immunosuppressive response facilitating carcinogenesis (Figure 1).

#### 3.3.2. Gut Microbiota Interaction with Immune and Nervous Systems in HCC

**Figure 2:**
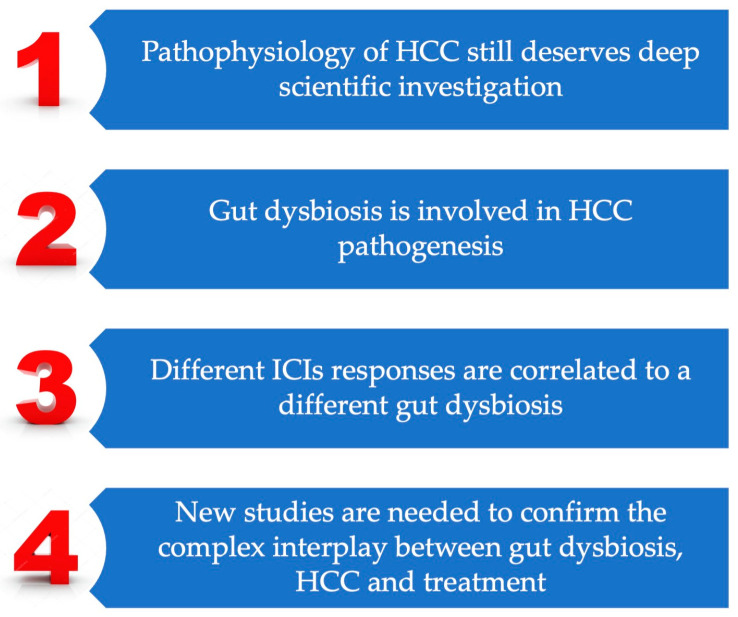
Key conclusive points for take-home messages.

The cornerstone of tumor progression is based on hepatic immunosuppression [61]. In fact, the liver is an immune-tolerant organ, physiologically exposed to gut-derived antigens, and it dampens inflammation [62]. This is the perfect landscape for rapid tumor growth and results in a limited efficacy of immunotherapy [63]. More in detail, there are both direct vagal nerve–tumor signaling [64] and “indirect” neuro-immune circuits that affect tumor growth [65]. Cholinergic vagal activity is able to affect gastrointestinal and extra-gastrointestinal cancer progression [66,67]. Within the liver, the vagal nerve has been shown to play a role in the prognosis of liver cancers [68]. Interestingly, cholinergic nerve density correlates with poor HCC outcome [69]. Furthermore, retrospective data from a large cohort of patients undergoing truncal vagotomy in the context of surgery for peptic ulcer had a decreased risk of liver and biliary cancers vs. those non-denervated [11]. Very recently, Bauer et al. [70] showed how surgical hepatic branch vagotomy in a mouse model correlated with reduced liver tumor development. Specifically, livers under vagotomy had an increased inflammatory state. Therefore, this evaluation reinforced the hypothesis on the existence of a cholinergic anti-inflammatory arc [71]. Indeed, there was a peculiar acetylcholine (ACh) regulation on different CD8+ T cell subsets: ACh release impairs CD8+ T cell inflammation, cytotoxicity, and tumor-mediated killing. On the other hand, the genetic deletion of AChR Chrm3 on CD8+ T cells reduced HCC tumor growth. More interestingly, mice with hepatic vagotomy presented with reduced cancer fatigue and anxiety responses vs. those with sham vagotomy. These altered behaviors significantly correlated with gut dysbiosis. Finally, HCC microbiota transplantation regulated hepatic anti-tumor immunity via vagal nerves vs. sham controls. Thus, we can assume a promising therapeutic impact for newer neuroimmune-directed therapy in liver cancer (Figure 2).

### 3.4. Perspectives on Gut Microbiota Modulation in the Context of Liver Cancer

There are several options for gut microbiota modulation in the context of liver cancer. Promising evidence supports the use of the Mediterranean diet to prevent and slow down liver cirrhosis progression. Moreover, this was associated with a reduced incidence of HCC [72]. Mediterranean diet administration significantly re-establishes the gut microbiota composition, leading to a higher abundance of genera such as Lactobacillus, Bifidobacterium, and Faecalibacterium [73]. However, there are some uncertainties regarding the Mediterranean diet. For example, its high fiber content can interact with BA concentration in the context of HCC pathogenesis [23,24]. Indeed, in the presence of gut dysbiosis, high BAs, and hyperbilirubinemia, administration of the Mediterranean diet may benefit from a low fermentable fiber concentration. From a pathophysiological point of view, there is growing evidence from observational investigations of the inverse relationship between the consumption of monounsaturated and omega-3 polyunsaturated fatty acids (namely and respectively, MUFAs and ω-3 PUFAs) and HCC prevalence [74]. Thus, MUFAs and ω-3 PUFAs can be protective through gut microbiome modulation, reducing intestinal mucosa inflammation and preventing HCC pathogenesis [75,76] (Table 3).

Among antibiotics active against Gram-positive bacteria, vancomycin can inhibit HCC development. Interestingly, this was accompanied by inhibition of the emergence of senescent hepatic stellate cells (HSCs) [77]. Within the experimental setup used in this study, the depletion of intestinal bacterial strains of Clostridium cluster XI and XIVa was included, essential for the overproduction of DCA. The latter is responsible for DNA damage, leading to the rise in senescent HSCs. In another cholestatic murine model of HCC, mice were fed a high inulin diet and treated with vancomycin. There was a significant reduction in HCC progression attributable to a reduced number of secondary BAs biosynthesized by the dysbiotic gut microbiota [78]. Furthermore, in a murine HCC model, an antibiotic cocktail of vancomycin, neomycin, and primaxin was associated, due to MYC overexpression, with a significant reduction in the number and size of HCC nodules vs. the control group [22,23]. Immunologically, the mixture used brought a selective pressure towards hepatic C-X-X motif receptor 6+ NKT cells spread within the liver. Unfortunately, data from human studies showed contrasting findings. However, fortunately, antibiotic use in HCC patients is an important biomarker for assessing the treatment outcome of patients under ICIs and tyrosine kinase inhibitors. For example, almost 4100 HCC patients belonging to nine multicenter clinical trials showed exposure to antibiotic treatment to be associated with worse treatment outcomes [79]. Similarly, data from 395 HCC patients in Hong Kong receiving ICIs confirmed the concurrent antibiotic use to correlate with higher mortality rates in patients with an advanced disease stage [80]. These findings can be explained by the disruption of the gut–liver axis. However, more investigations are warranted in the field. Probiotics are alive organisms beneficially affecting host health [81]. Despite the well-known and evidence-based mechanism of their action preventing the development of chronic liver disease complications like HCC, they can also manipulate the binding and absorption of carcinogens. For example, targeting aflatoxins such as hepatocarcinogen, patients treated with a multistrain probiotic (*Lactobacillus* and *Propionibacterium* spp.) had a lower urinary level of the toxins [82]. Moreover, animal studies explained that the strains are able to bind aflatoxins [83]. Indeed, we cannot forget the immunomodulant mechanism of action involving the gut–liver axis. Mice fed a novel probiotic mixture (Lactobacillus species, Escherichia coli, and heat-inactivated VSL#3) resulted in Th17 cell migration into liver cancer. Interestingly, this led to a decrease in tumor weight and size. In addition, there was a downregulation of angiogenic factors [84]. Accordingly, the gut microbiota showed a higher abundance of Butyricimonas and Prevotella genera. Using a rat model of HCC, VSL#3 alone significantly reduced intestinal permeability and plasma levels of LPS. Similarly, there was a significant reduction in HCC weight and size in treated animals vs. controls [85]. The direct modulation of a living “organ” as the human gut microbiota in patients with liver cancer can be obtained with fecal microbiota transplantation (FMT). In this regard, FMT has been shown to effectively attenuate high-fat-diet-induced steatohepatitis and further potential liver cirrhosis and/or HCC development [86]. In addition to this solid evidence, there are promising data on the adjuvant role of FMT in non-HCC-ICI-treated patients. More in detail, in melanoma patients, FMT from responders to PD-1 blockade delivered to non-responders resulted in an improved response to treatment [87]. Immunologically, treatment-responders showed an increased gut infiltration of antigen-presenting cells and tumor-infiltrating lymphocytes [88]. Interestingly and specifically, the genus Lachnoclostridium was associated with an enrichment of UDCA, tauro-UDCA, UCA, and MDCA. They are secondary BAs associated with the response to ICI therapy in HCC [89]. Accordingly, Akkermansia muciniphila’s gut microbiota enrichment has been linked to ICI treatment-response in a variety of solid cancers including HCC [90] (Table 4).

## 4. Conclusions

The pathophysiology of HCC is a topic that still requires investigation. Currently, only a few genetic mutations can be linked to HCC. The discovery of weak immunosurveillance within the liver has led to a shift in cancer treatment from anti-angiogenetic to novel immunotherapy. Hepatic immune response is regulated by the gut microbiota also through BA and SCFA metabolism [93,94].

Different gut dysbioses characterize different ICI responses. Moreover, different gut dysbioses characterize different liver disease stages (e.g., HCC in liver cirrhosis vs. non-liver cirrhosis). Gut dysbiosis is a hallmark of the etiology of different liver diseases.

Thus, gut dysbiosis can be a useful biomarker for HCC diagnosis and treatment-response monitoring in the frame of “personalized” medicine.

Finally, gut dysbiosis modulation with diet, pre-/probiotics and FMT show promising results in their ability to drastically change the natural course of HCC [93,94].

The key conclusive points are summarized in Figure 2 as take-home messages.

## Figures and Tables

**Table 1:** Challenges and promising results regarding medical and therapeutic needs in HCC patients.

Issues of Current Available Treatments	Subsequent Main Needs in HCC Treatment	Promising Results
- Extremely variable treatment-response of HCC patients; - High rate of advanced- vs. early-stage HCC patients; - Only a few known genetic mutations for HCC (e.g., TERT, CTNNB1, TP53); - Lack of effective biomarker(s).	- Patients’ HCC stage stratification; - Available biomarker(s) to allow early HCC patient recognition; - Screening for highly mutated genetic targets; developing new pathways to target existing mutations; - Biomarker(s) for diagnosis and monitoring of patients’ treatment-response.	- Use of nonsteroidal anti-inflammatory agents; attempts to target the WNT/beta-catenin pathway; - Silencing TERT expression with antisense oligonucelotides; overexpression of wild-type AXIN1; attempts to target the p53 pathway; - New HCC biomarker(s) (e.g., gut microbiota).

Abbreviations: HCC: hepatocellular carcinoma; TERT: telomerase reverse transcriptase; WNT: wingless/integrated; CTNNB1: catenin beta-1; TP53: tumor protein; AXIN1: axis inhibition protein 1.

**Table 2:** Comparative gut dysbiosis and liver conditions associated with and without liver cancer.

Liver Condition	Bacterial Dysbiosis	Comparison Group	Reference
HCC in liver cirrhosis patients	*Escherichia coli ↑*	vs. HCC patients without liver cirrhosis	[30]
HCC in liver cirrhosis patients	Phylum *Actinobacteria ↓* *Verrucomicrobia ↑* Genus *Gemmiger* and *Parabacteroides ↓* *Alistipes*, *Phascolarctobacterium,* and *Ruminococcus ↑* *Klebsiella* and *Haemophilus ↓*	vs. HCC patients without liver cirrhosis	[32]
HCC in MASH-related liver cirrhosis	*Bacteroides* and *Ruminococcaceae ↑* *Bifidobacterium ↓*	vs. non-MASH-related liver cirrhosis	[32]
HBV-related HCC	*Escherichia-Shigella*, *Enterococcus ↑*	vs. non-HBV-related HCC	[37]
HBV and HCV-related HCC	*Faecalibacterium*, *Ruminococcus*, *Ruminoclostridium ↑*	vs. non-HBV and HCV-related HCC	[36]

Abbreviations: ↑: increased abundance; ↓: decreased abundance; HCC: hepatocellular carcinoma; MASH: metabolic-dysfunction-associated steatohepatitis; HBV: hepatitis B virus; HCV: hepatitis C virus.

**Table 3:** Therapeutic approaches targeting the gut microbiota in liver cancer patients.

Therapeutic Approach	Mechanism(s) of Action	Impact on HCC	Reference
Mediterranean diet (prospective study; prospective parallel group; RCT crossover trial)	Increased gut microbiota diversity, resulting in reduced LPS concentration in compensated liver cirrhosis patients vs. uncompensated ones (*p* = 0.013).	Reduced HCC prevalence.	[23,24,69,70]
Monounsaturated fatty acids (MUFAs) and omega-3 polyunsaturated fatty acids (PUFAs) (crossover multicentric study using multivariable calibrated models; retrospective case–control study)	Increased gut microbiota diversity, resulting in reduced inflammatory response.	Reduced HCC risk of occurrence: in multivariable calibrated models, there is a statistically significant inverse association between total fat intake and risk of HCC (per 10 g/day, HR = 0.80, 95% CI: 0.65–0.99), mainly driven by MUFA (per 5 g/day, HR = 0.71, 95% CI: 0.55–0.92) rather than PUFA (per 5 g/day, HR = 0.92, 95% CI: 0.68–1.25).	[71,72,73]
Antibiotics (animal model and prospective observational study)	Reduced abundance of commensal bacteria (e.g., *Clostridium* cluster XI and XIVa), reduced cluster of secondary BAs (e.g., DCA), resulting in reduced concentration of senescent HSCs and increased concentration of hepatic NKT cell infiltration; disruption of the gut–liver axis due to increased dysbiosis.	Reduced tumor growth in animal models; reduced response to ICIs, resulting in higher mortality in humans: comparing the propensity score of 56 antibiotic users with 99 non-users showed that concurrent antibiotic use with ICI was associated with higher cancer-related (aHR: 1.66; 95% CI: 1.08–2.54) and all-cause mortality (aHR: 1.63; 95% CI: 1.17–2.28).	[74,75,76,77]
Probiotics (prospective parallel group comparing single vs. multistrain; prospective RCT)	Reduced weight and size of HCC.	Increased abundance of beneficial species/reduced abundance of pathogenic species, resulting in reduced intestinal inflammation, reduced intestinal permeability, and lower circulating LPS levels. Removal of carcinogens through binding by probiotics: ninety healthy young Chinese men were randomized into the following: one group receiving *Lactobacillus rhamnosus LC705* and *Propionibacterium freudenreichii* subsp. *shermanii* strains 2 times/d for 5 weeks; one group receiving placebo. There was a statistically significant decrease in the aflatoxin-N(7)-guanine urine concentration in the probiotic-treated group only (36% at week 3 and 55% at week 5 follow-up).	[78,79,80,81,82]
Fecal microbiota transplantation (prospective exploratory study; phase 1 clinical trial)	Reduced hepatic inflammation and HCC risk. Increased effectiveness of ICI treatment, reduced HCC growth.	Gut dysbiosis modulation resulting in increased immune response vs. tumor: out of 10 patients with anti-PD-1-refractory metastatic melanoma, there was clinical response in 3 patients.	[83,84,85,86,87]

Abbreviations: LPS: lipopolysaccharide; HCC: hepatocellular carcinoma; BA: bile acid; DCA: deoxycholic acid; ICIs: immune checkpoint inhibitors; RCT: randomized controlled trial; HR: hazard ratio; CI: confidence index.

**Table 4:** Therapeutic approaches targeting the gut microbiota in liver cancer animal models.

Animal Model	Treatment Used	Impact on HCC	Reference
Inulin-fed HCC in T5KO mice	Vancomycin	Gut microbiota modulation: *Bifidobacteria*, *Lachnospiraceae*, *Clostridium cluster XIVa*, and *Ruminococcaceae ↓.* Secondary BA concentration reduction significantly correlated with gut microbiota vancomycin-related modulation.	[77]
MYC transgenic spontaneous HCC in mice	Antibiotics’ cocktail (namely, vancomycin, neomycin, and primaxin)	Gut microbiota modulation: *Bacteroidales ↓*, *Verrucomicrobiales ↑*. In antibiotic-treated MYC mice, there was a significantly reduced number of HCC nodules.	[45]
Hepa 1–6 (mouse hepatoma cell line) injected HCC in C57BL6/N mice	Prohep (probiotics mixture)	Significant gut microbiota modulation: *Prevotella*, *Oscillibacter*, *Akkermansia muciniphila*, *Bacteroides fragilis*, *Alistipes*, *Paraprevotella*, *Mucispirillum ↑.* Gut microbiota modulation significantly correlated with decreased tumor size and weight by 40% vs. control group. *Prevotella* and *Oscillibacter* anti-inflammatory cytokines inhibited Th17 polarization and increased the development of anti-inflammatory Treg/Tr1 cells within the gut.	[83]
Diethylnitrosamine (DEN)-induced HCC in rats	VSL#3 (probiotics’ mixture including 8 bacterial species)	*Escherichia coli*, *Bacteroides fragilis group,* and *gram-bacteria ↓*; Reduced incidence and number of HCC nodules in treated animals.	[91]
Bcr-Abl-transfected No (BaF3 cells) tumor in mice	Inulin-type fructans	No significant gut dysbiosis modulation; lowered hepatic BaF3 cellular invasion and inflammation and increased portal propionate concentration.	[92]

Abbreviations: HCC: hepatocellular carcinoma; ↑: increased abundance; ↓: decreased abundance; BA: bile acid; MYC: myelocytomatosis oncogene, T5KO: Flagellin receptor TLR5-deficient (mice).

## Data Availability

Data from the contribution are available online on PubMed and on the main Gastroenterology and Hepatology congress websites.
